# COVID-19 infection manifesting as a severe gastroparesis flare: A case report

**DOI:** 10.1097/MD.0000000000025467

**Published:** 2021-04-09

**Authors:** Jun Song, Rajiv Bhuta, Kamal Baig, Henry P. Parkman, Zubair Malik

**Affiliations:** a Temple University Hospital, Department of Medicine; b Section of Gastroenterology and Hepatology, Department of Medicine, Temple University School of Medicine, Philadelphia, PA, USA.

**Keywords:** coronavirus, COVID-19, gastrointestinal symptoms, gastroparesis

## Abstract

**Rationale:**

Coronavirus disease 2019 (COVID-19) is a disease caused by infection with severe acute respiratory syndrome coronavirus 2 (SARS-CoV-2), which commonly presents with symptoms including fever, cough, and dyspnea. More recently, however, some patients have tested positive for COVID-19 after developing gastrointestinal (GI) symptoms either solely or in conjunction with respiratory symptoms. This may be due to SARS-CoV-2 infection of the GI tract. In patients with chronic GI illnesses, COVID-19 may initially present as a flare of their underlying GI conditions as viruses have historically been implicated in exacerbations of GI disorders, including gastroparesis.

**Patient concerns:**

We report a case of a 37-year-old female with a history of diabetic gastroparesis who presented to the Emergency Department (ED) with nausea and vomiting similar to her gastroparesis flares.

**Diagnoses:**

Her symptoms in the ED failed to improve with fluids and anti-emetic medications. After developing a fever, she was tested and found to be positive for COVID-19.

**Interventions:**

She was started on antibiotic, steroid, and antiviral medications.

**Outcomes:**

Her symptoms improved, her fever defervesced on day 4 of hospitalization, and she was discharged on day 5 of hospitalization. The patient reported symptom improvement at a follow-up outpatient gastroenterology visit 2 months after hospitalization.

**Lessons:**

To the best of our knowledge, at the present time, this is the first report of a patient with COVID-19 presenting with signs and symptoms of a gastroparesis flare. This case illustrates that COVID-19 may present in an exacerbation of symptoms of an underlying disorder, such as a severe gastroparesis flare, in a patient with underlying gastroparesis. Initial presentation of these patients manifesting as a flare of their chronic GI disease, more severe than usual, should prompt an index of suspicion for COVID-19.

## 1 Introduction

Coronavirus disease 2019 (COVID-19) is a disease caused by infection with severe acute respiratory syndrome coronavirus 2 (SARS-CoV-2). Since the initiation of the COVID-19 pandemic, patients with SARS-CoV-2 infections have primarily presented with respiratory symptoms as the virus mainly targets the respiratory tract.^[2]^ This predilection for the respiratory system may, in part, be due to pre-existing pulmonary comorbidities.^[3]^ However, a minority of patients have now been documented to initially exhibit gastrointestinal (GI) symptoms including nausea, vomiting, and diarrhea.^[4,5]^ The etiology for these GI symptoms secondary to SARS-CoV-2 infection is currently not well understood.^[6]^ It is possible that chronic GI disorders may similarly predispose patients to primary GI infections from SARS-CoV-2 and manifest COVID-19 as a flare of their underlying disease. Herein, we report a case of a 37-year-old female with diabetic gastroparesis who tested positive for COVID-19 after she presented to the Emergency Department (ED) for intractable emesis, thought to be a flare of her gastroparesis.

## 2 Case report/case presentation

**Figure 1:**
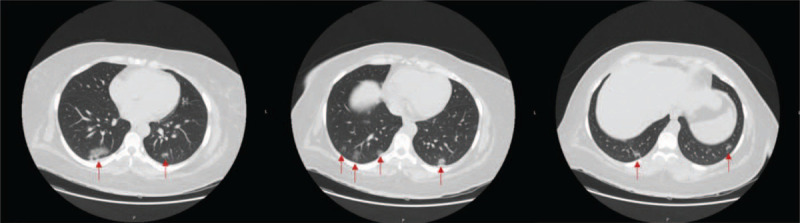
CT Chest shows multifocal lower lung predominant peripheral bronchocentric ground glass opacities (red arrows) compatible with viral/atypical pneumonia.

A 37-year-old female with a history of diabetic gastroparesis presented with 2 days of intractable nausea and vomiting. She was diagnosed with type II diabetes mellitus prior to 2017 at an outside institution, now treated with insulin. Prior upper endoscopy was negative and prior gastric emptying study was significant for 87% retention of radiolabeled meal at two hours (normal < 60%) and 77% at four hours (normal < 10%). At presentation in the ED, the emesis was non-bloody, non-bilious, and associated with mild epigastric pain. Review of systems was negative for fevers or respiratory symptoms. Vital signs were within normal range. Her home medication list included atorvastatin, insulin, gabapentin, metformin, ondansetron, and pantoprazole. The cause of her symptoms was initially attributed to a gastroparesis flare in the setting of medication non-adherence as she had multiple ED visits in the past with similar presentations controlled with pharmacological treatment. She was discharged after a trial of metoclopramide administration. She returned the next day with worsening nausea and vomiting, which were described as significantly increased from her baseline gastroparesis flares, refractory to pharmacological therapy. Vitals were significant for a temperature of 100.4°F. Laboratory tests were notable for a Hemoglobin A1c (HgbA1c) of 7.2% from 11.7% one-year prior (normal: 4.7–6.4%), thyroid stimulating hormone (TSH) level of 1.10 m[iU]/L (normal: 0.40–4.50 m[iU]/L), and initial glucose level of 181 mg/dL. She also had lymphopenia with a white blood cell count of 3.4 K/mm^3^ (normal: 4.0–11.0 K/mm^3^), which was reduced by about 5 K/mm^3^ from her baseline. Six hours after initial triage, she developed a fever of 101.4°F. A chest x-ray and the CT Abdomen and Pelvis were unremarkable. The CT chest (Fig. 1), however, showed multi-focal ground glass opacities predominantly in the lung bases compatible with a viral/atypical pneumonia. A nasopharyngeal COVID-19 test was obtained, antibiotics (azithromycin and ceftriaxone) were started empirically, and she was admitted to the hospital. On day 2 of admission, the gastroenterology service was consulted for the management of her gastroparesis flare and recommended continuing pantoprazole, changing the administration of ondansetron and metoclopramide to standing medications, and starting mirtazapine. During this time, her COVID-19 test resulted positive. In addition to antibiotics, she was started on steroids and entered into a Remdesivir clinical trial. Her aspartate aminotransferase (AST) values had an upward trend from Day 2 to Day 3: 21, 21, 40, and 50 U/L (normal range: 15–37 U/L). Her glucose levels ranged from 154 to 301 mg/dL (average: 197.7 mg/dL) throughout her admission. She reported symptom improvement by day 3, defervesced by day 4, and was discharged on day 5. She was instructed to continue taking mirtazapine and ondansetron to manage her gastroparesis as well as antibiotics, steroids, and Remdesivir for COVID-19. She was evaluated 2 months later in the outpatient gastroenterology office for follow-up. She reported further improvement in symptoms after being discharged and subsequent flares of her gastroparesis were less severe and less frequent. Furthermore, her AST values normalized to 18 U/L.

## 3 Discussion and conclusion

This case report illustrates that COVID-19 infection can present as a flare of gastroparesis. The important take home message is that in patients with an exacerbation of a chronic illness, such as gastroparesis, one should consider COVID-19 as a potential etiology for the worsening of a previously stable chronic disorder.

The majority of patients with COVID-19 have currently presented with respiratory symptoms.^[2,7]^ However, more patients are starting to present with GI symptoms either solely or in conjunction with pulmonary manifestations.^[4,8]^ This involvement with the GI tract has been reflected in reports of patients testing positive for infection in fecal samples as well as abnormal liver function tests, which could explain the rise in AST in our patient.^[9,10]^ Immunofluorescence studies by Xiao et al have also shown that the SARS-CoV-2 ACE2 protein cell receptor is expressed in the glandular cells of gastric, duodenal, and rectal epithelia potentially allowing viral entry into these cells.^[11]^ These are the same cell receptors found in the bronchial epithelial cells in the lower respiratory tract responsible for SARS-CoV-2 infection in the respiratory system.^[12]^ Other coronaviruses that have also caused respiratory syndromes such as Middle East Respiratory Syndrome Coronavirus and SARS-CoV-1 have similarly been found to infect intestinal cells and spread through a fecal-oral route.^[13–15]^ Taken together, it may be possible that the GI tract is also serving as a nidus for SARS-CoV-2 infection. Though, the predilection of SARS-CoV-2 for either the pulmonary system or the GI tract is not well understood despite similar infection mechanisms.

In epidemiologic studies, comorbidities of patients may be a risk factor for infection with SARS-CoV-2. In a meta-analysis including 1576 patients, respiratory system diseases were among the most prevalent comorbidities affecting those with COVID-19 presenting primarily with respiratory symptoms.^[16]^ Furthermore, Leung et al found that patients with Chronic Obstructive Pulmonary Disease (COPD) have upregulated airway expression of the ACE2 protein cell receptor, which may increase the risk of patients with COPD to SARS-CoV-2 infection.^[12]^ This suggests that comorbidities may play a role in the pathogenesis of SARS-CoV-2. In a recent study of 116 patients with COVID-19 who initially presented with cough, fever, and dyspnea as the main presenting symptoms, nearly a third of the patients were reported to develop GI symptoms later in the course of their infection.^[6]^ In this series, 20.7% of patients had chronic pulmonary disorders and 26.1% had a smoking history, while there were no patients with underlying GI disorders and only 2.8% had chronic liver disease. The pulmonary comorbidities may have served as a risk factor for initial pulmonary infection in this cohort of patients. The infection may have subsequently propagated to the GI tract, demonstrated by the delay in GI symptoms. Although the pathogenesis of SARS-CoV-2 infection to different organs is yet to be fully understood, SARS-CoV-2 RNA has previously been detected in the blood of patients confirmed to have COVID-19.^[17]^ This suggests the possibility for inter-organ dissemination of SARS-CoV-2 secondary to viremia after initial inoculation given the presence of SARS-CoV-2 receptors on multiple organ systems.^[18]^ This would suggest that comorbidities may play a key role in the initial pathogenesis of SARS-CoV-2. In the context of our patient, this may explain why she developed evidence of pulmonary disease on imaging despite presenting solely with GI symptoms. Her underlying gastroparesis may have predisposed her to infection with SARS-CoV-2 in the GI tract as evidenced by her symptom severity and uptrending liver enzyme values.^[19]^ Her infection may have subsequently spread to her lungs as manifested by the multi-focal ground glass opacities found on imaging. Further research is needed to determine if patients are at higher risk for SARS-CoV-2 infection in the GI tract with certain chronic GI conditions.

Though it is possible that her intractable nausea and vomiting were entirely independent of COVID-19, an exacerbation of gastroparesis from SARS-CoV-2 is the likely scenario, as viruses have been implicated in the pathogenesis of gastroparesis.^[20,21]^ In one study investigating the etiology of the disorder, 23% of patients with gastroparesis have been described as having postinfectious gastroparesis from a prior viral infection.^[22]^ The proposed mechanism by which viruses can induce gastroparesis is through an acute injury to either the neural innervation of the stomach or the interstitial cells of Cajal in the stomach causing delayed gastric emptying.^[23]^ Similarly, infections have also been implicated in worsening of other chronic GI disorders, such as inflammatory bowel disease (IBD).^[24]^ Kim et al found that patients with ulcerative colitis (UC) with concomitant cytomegalovirus (CMV) infections had a higher frequency of IBD-related hospitalizations compared to patients with UC without CMV infections.^[25]^ This increased morbidity in the cohort with CMV infections may be due to the ability of CMV proteins to enhance pro-inflammatory cytokines to worsen the inflammation causing a flare of their UC more severe than baseline.^[26]^ Therefore, it is reasonable to suspect that a virus like SARS-CoV-2 could potentially exacerbate an underlying GI disorder causing a flare of the disease during the initial presentation of COVID-19. Beyond infectious causes, other etiologies found to induce gastroparesis flares include worsening diabetes, hypothyroidism, surgery, medications, and recreational drug use.^[27]^ Our patient had improved glycemic control (HgbA1c 7.2% from 11.7% a year prior) and normal TSH levels. She did not have any recent surgeries, take any new medications, or use recreational drugs (including opiates). Therefore, infection with SARS-CoV-2 is the likely etiology for the gastroparesis flare in our patient.

To the best of our knowledge, at the present time, this is the first case of COVID-19 initially manifesting with signs and symptoms of a gastroparesis flare. Our patient had significant improvement in her symptoms after treatment of COVID-19 with antibiotic, steroid, and antiviral medications. It is important to note, however, that azithromycin has been found to have pro-motility effects in the smooth muscle of the gut.^[28]^ Therefore, the use of azithromycin in our patient may have served a dual purpose by both treating her pulmonary pathology seen on imaging and exerting prokinetic effects in the GI tract. It is unclear how much of a direct role azithromycin served in providing symptomatic relief in our patient as research in macrolide therapy for treatment of gastroparesis is currently limited. This unique case illustrates that COVID-19 may present with exacerbated symptoms of an underlying disorder, such as a severe gastroparesis flare in a patient with underlying gastroparesis. COVID-19 should be suspected in patients presenting in a flare, more severe than usual, of their underlying medical disease.

## Author contributions

JS reviewed the patient's hospital course, wrote and revised the manuscript. RB, KB, and HP revised the manuscript. ZM revised the manuscript and is the article guarantor.

**Conceptualization:** Jun Song.

**Investigation:** Jun Song.

**Writing – original draft:** Jun Song.

**Writing – review & editing:** Jun Song, Rajiv Bhuta, Kamal Baig, Henry P. Parkman, Zubair Malik.
